# Maternal exposure to titanium dioxide nanoparticles disrupts ultrasonic vocalization development in mouse offspring

**DOI:** 10.1186/s12989-026-00668-7

**Published:** 2026-04-02

**Authors:** Marie Boulain, Taner Aktas, Gilles Courtand, Grégory Barrière, Muriel Thoby-Brisson, Didier Morin, Laurent Juvin

**Affiliations:** Univ. Bordeaux, CNRS, INCIA, UMR 5287, Bordeaux, F-33000 France

**Keywords:** Nanoparticles, TiO2, Mouse, Vocalization, Maternal exposure

## Abstract

**Background:**

Early neurodevelopment is a critical period during which environmental exposures can have lasting effects on brain function and behavior. One key indicator of early neurodevelopmental integrity in rodents is the production of neonatal ultrasonic vocalizations (USVs), which are essential for maternal-offspring communication. Given the widespread use of titanium dioxide nanoparticles (TiO2NPs) in food and consumer products, there is growing concern that perinatal exposure to these particles may interfere with normal neurodevelopment. However, the effects of TiO2NPs exposure on USV production remain poorly investigated.

**Results:**

In the present study, pregnant mice were orally exposed to TiO2NPs (200 µg/g) from conception to weaning, and their offspring underwent a maternal separation test to assess USVs between postnatal day P2 and P13. TiO2NP-exposed pups exhibited a significant reduction in the number of USVs at P6-7, accompanied by a delayed peak vocalization period. This reduction was primarily attributable to shorter vocalization series rather than fewer isolated calls. Additionally, acoustic analysis revealed that pups emitted two types of USVs, simple and complex, both of which were significantly reduced in number at P6-7 in the exposed group. Fast Fourier transform (FFT)-based analysis showed that complex USVs had a lower mean frequency, while both call types exhibited increased variability in mean frequency. Furthermore, TiO2NP-exposed pups displayed alterations in USV syntax, including a lower proportion of simple USVs and disrupted developmental maturation of call structure. Electrophysiological recordings revealed that the intermediate reticular oscillator (iRO), a key brainstem center involved in vocalization control, exhibited reduced excitability and an increased activity variability in exposed pups, suggesting that nanoparticle exposure compromises vocal motor regulation at the neural level. Lastly, playback experiments demonstrated that USVs from TiO2NP-exposed pups failed to elicit appropriate maternal attraction, indicating impaired communicative effectiveness.

**Conclusions:**

Perinatal exposure to TiO2NPs disrupts the normal development of USVs, impairing both vocalization patterns and neural excitability of the iRO. These changes may contribute to altered maternal-offspring interactions and highlight the potential neurodevelopmental risks of early-life TiO2NPs exposure. Given the widespread presence of TiO2NPs in consumer products, further research is necessary to assess their long-term consequences on neural circuits underlying communication and social behavior.

**Supplementary Information:**

The online version contains supplementary material available at 10.1186/s12989-026-00668-7.

## Introduction

 Neonatal communication plays a fundamental role in early development, particularly in altricial species where survival heavily depends on maternal care. In rodents, ultrasonic vocalizations (USVs) emitted by pups serve as crucial signals that elicit maternal responses, ensuring warmth, nourishment, and protection [1, 2]. The development and organization of USVs follow a well-defined trajectory, characterized by changes in vocalization number, syntax, and acoustic properties throughout early postnatal stages [3, 4]. Disruptions in USV production can indicate underlying alterations in neural circuits controlling vocal behavior and may have significant implications for social and cognitive development [5, 6].

Among the numerous environmental factors that influence early neurodevelopment, exposure to nanoparticles (NPs) has emerged as a growing concern due to their widespread presence in food, cosmetics, and pharmaceuticals [7, 8]. Titanium dioxide nanoparticles (TiO2NPs) are one of the most commonly used nanomaterials, with potential risks associated with prenatal and postnatal exposure. Previous studies have indicated that TiO2NPs can cross biological barriers, accumulate in the brain, and induce oxidative stress, neuroinflammation, and alterations in neurotransmitter systems [9–16]. Additionally, maternal exposure to TiO2NPs alters pup development, affecting both body weight and size as well as respiratory activity [17–19]. Finally, TiO2NP exposure has been shown to impact the activity and development of brainstem respiratory centers [17]. USV production is closely linked to respiratory function and is generated by neural centers located in the brainstem, which interact strongly with respiratory control circuits [20–22]. Given this interaction, any factor disrupting respiratory activity and its neural control could potentially impair USV production and, consequently, maternal-infant communication.

In this study, we investigated the impact of perinatal exposure to TiO2NPs on USV production and organization in neonatal mice. We hypothesized that TiO2NPs exposure could alter the developmental trajectory of USV emission, affecting the number, structure, and communicative efficacy of vocalizations. To test this, we exposed pregnant mice to TiO2NPs via dietary administration and assessed USV production in their offspring during the early postnatal period. Additionally, we examined potential alterations in the neural circuits responsible for USV generation, focusing on the intermediate Reticular Oscillator (iRO), a key brainstem region implicated in vocal patterning [20]. Lastly, we evaluated the behavioral consequences of altered USV production by conducting playback experiments to assess maternal responsiveness to pup calls. By elucidating the effects of TiO2NPs exposure on early vocal communication and its neural basis, this study aims to provide novel insights into how environmental factors can impact neurodevelopmental processes essential for social behavior.

## Materials and methods

### Ethical approval

All procedures were conducted in accordance with the local ethics committee of the University of Bordeaux (APAFIS#11978-2017103012063751) and the European Communities Council Directive (2010/63/EU). Experiments were performed on OF1 mice that were obtained from the CIRCE (Behavioral Engineering Centre) facility of Bordeaux Neurocampus. Newborns had full access to their mother’s milk, and the mothers were fed *ad libitum* with full access to water. Every effort was made to minimize suffering and the number of animals used.

### Maternal exposure to TiO2NPs

**Fig. 1:**
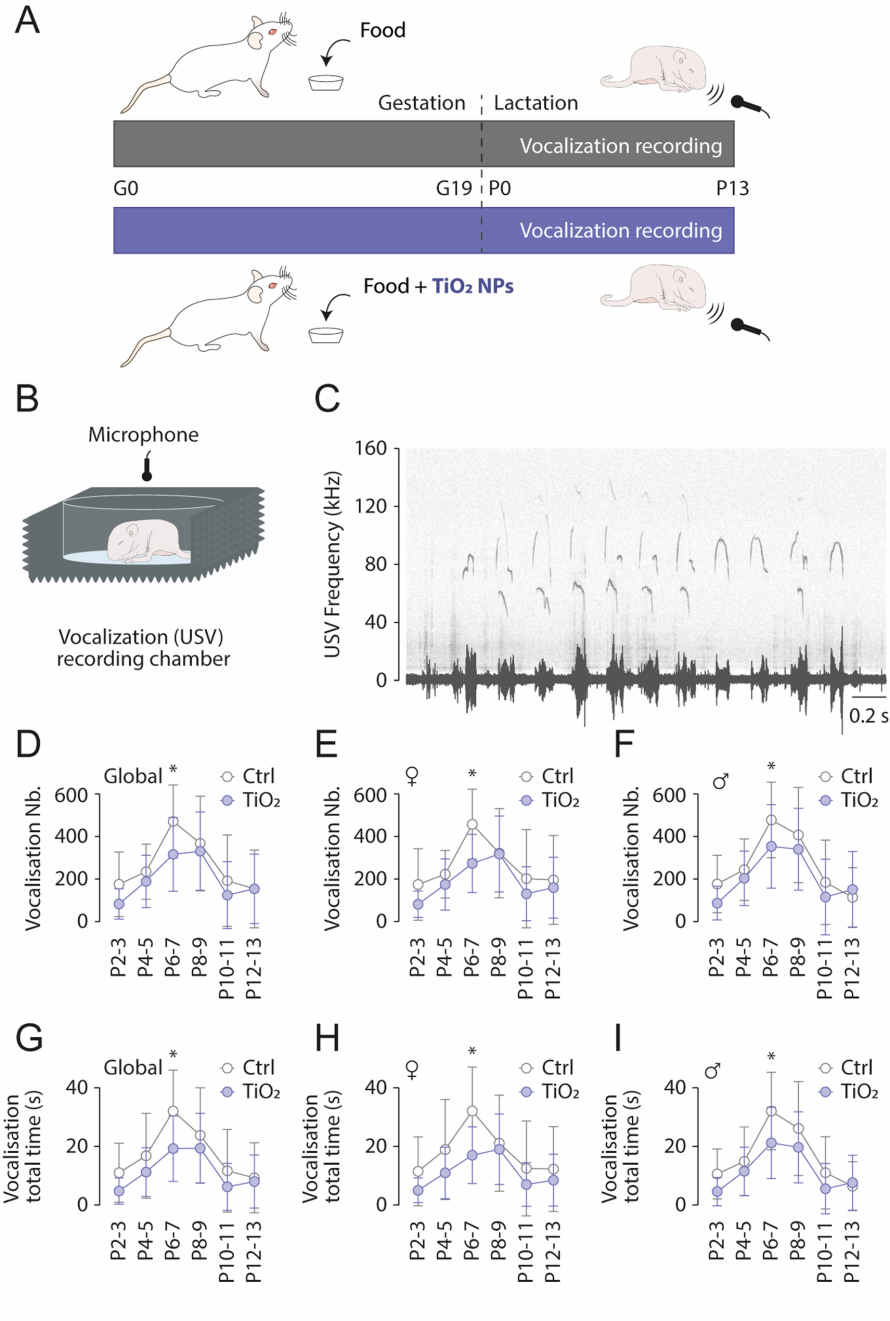
Perinatal exposure to TiO2NPs reduces offspring vocalization. **A** Schematic representation of the TiO2NPs exposure protocol during the perinatal period. **B** Schematic representation of the ultrasonic vocalization (USV) recording setup. **C** Audio recording of pup USVs: the lower trace represents a raw recording, while the upper graph shows the corresponding sonogram. **D–F** Scatter plots illustrating the number of USVs produced by pups during the 5-minute maternal separation test across the P2-P13 period: global (**D**), female pups (**E**), and male pups (**F**). **G–I** Similar representation for the total vocalization duration. Number of female Control pups: 14; Number of male Control pups: 16; Number of female TiO2NPs pups: 12; Number of male TiO2NPs pups: 12

To generate the F1 offspring, F0 pregnant mice ingested a daily dose of TiO2NPs (200 µg/g, *n* = 17) that was mixed with chocolate spread (500 µg/g), and administered by voluntary food intake, from the first gestational day (determined by the presence of a vaginal plug the morning following the mating night) until weaning (Fig. 1A). A second group of F0 dams received a daily dose of chocolate spread (500 µg/g, *n* = 19). Pregnant mice were weighed daily, as were newborns from birth until the 11th day of life. TiO2NPs were purchased from Sigma-Aldrich (ref. 718467).

### USV recording

To measure ultrasonic vocalizations (USV) of newborns, animals were placed for 5 min in a soundproof chamber lined with acoustic foam and equipped with a Pettersson USB M500-384 ultrasound microphone (Pettersson). To prevent heat loss in the newborns, the chamber was placed on a heating pad, and the room temperature was maintained at 24 °C throughout the recording period.

Ultrasonic vocalizations were recorded using Audacity software at an acquisition frequency of 384 kHz.

Detection and analysis of USV were performed using a MATLAB script USVEG [23]. Fast Fourier Transform (FFT) was performed using Spike2 software (Cambridge Electronic Design).

### Brainstem slices

Animals (postnatal day 0 to 7) were deeply anesthetized with 4% isoflurane and subsequently decapitated. All preparations were dissected in an artificial CSF (aCSF) solution composed of (in mM): 120 NaCl, 8 KCl, 1.26 CaCl_2_, 1.5 MgCl_2_, 21 NaHCO_3_, 0.58 NaH_2_PO_4_, and 30 D-glucose, pH 7.4, maintained at 4 °C and continuously bubbled with a mixture of 95% O_2_ and 5% CO_2_. 550 μm thick transverse brainstem slices were obtained by serially sectioning in the rostral-to-caudal direction isolated hindbrains mounted in a low-melting-point agar block using a vibratome (Leica VS 1000). Slices containing the intermediate reticular oscillator (iRO) were placed in a recording chamber and superfused continuously with aCSF equilibrated with 95% O_2_/5% CO_2_, pH 7.4 at 30 °C. Of note, the rostral side of the slices containing the iRO were taken 100 to 200 μm caudal to the end of the facial nucleus, at the rostral end of the compact nucleus ambiguus [20].

### Electrophysiological recording

Extracellular recordings were obtained using borosilicate filamented glass electrodes (Harvard Apparatus, Germany), with the tip diameter visually adjusted to match the size of the recording area. Signals were amplified (10,000x) by differential AC amplifiers (low cutoff, 100 Hz; high cutoff, 1 kHz; model 1700, A-M Systems), digitized and acquired via a CED1401 interface, stored on a computer, and analyzed using Spike2 software (Cambridge Electronic Design).

### Playback experiments

A two-choice playback test was conducted using recordings of USVs emitted by either exposed or non-exposed 7-day-old (P7) pups. Non-exposed adult female mice with prior maternal experience were placed in the experimental setup (Fig. 8). The setup (60 × 45 cm) consisted of three equally sized chambers, with the USVs being played back in one of these three chambers using an Avisoft UltraSoundGate Player 116 H and an ultrasonic dynamic speaker (Avisoft Bioacoustics). Video recordings were made with a Raspberry Pi, and mice movements were detected and analyzed using the Mouse Track software (https://github.com/gillescourtand/Ultimate_Tracker).

### Statistical analysis

Group values were expressed as means +/- SD. Differences between means were analyzed using Prism 8.4.2 Software (GraphPad) and assessed by Student’s t-test, 2 Way ANOVA and Sidak’s multiple comparison tests. The stability of iRO oscillations was assessed by calculating the coefficient of variation of the iRO period over 120–180 s of activity, using Prism 8.4.2 Software (GraphPad) [24]. The coordination between the activities of left and right iROs was examined by means of cross-correlation analysis of ≥ 120 s of activity [24]. Cross-correlations coefficients between left and right iROs activities allowed estimation of coupling strength whereby values approaching 1 indicate synchrony, whereas values toward − 1 reflect burst alternation and finally a flat cross-correlogram at ∼0 indicates independent rhythms. Differences in mean values for each parameter were taken to be significant at *p* < 0.05.

## Results

### Perinatal exposure to TIO2NPs impairs offspring vocalization number

In the present study exposed females were given daily doses of P25 titanium dioxide nanoparticles (TIO2NPs) at 200 mg/kg (Fig. 1A), starting from the first day of gestation and continuing until the end of lactation and weaning [17, 19]. TiO2NPs used in the present study had been previously characterized in our earlier work, with a composition of 80% anatase and 20% rutile, an average particle size of 25 nm, and a zeta potential of -25 mV [17]. Using transmission electron microscopy in the same study, we further assessed particle size and polydispersity, revealing a mean diameter of 24 ± 8 nm, with sizes ranging from 5 to 50 nm. Typical adult exposure to titanium dioxide ranges from 0.3 to 3.8 mg per kilogram of body weight per day [25]. In our study, the daily dose administered was approximatively fifty times greater than this estimated exposure (Figure S1A). Nevertheless, when considering the full ~ 19-day gestation period in mice, the total amount of TiO2 nanoparticles received (i.e. 167 mg, Figure S1B) is still about five hundred times lower than the amount a woman would be expected to ingest over the course of an approximately 280-day pregnancy.

In pups, vocalization is a crucial function, as it helps trigger maternal care and prevent death in neonates, whose temperature maintenance is not yet functional and strictly depends upon mother support. To assess the production of ultrasonic vocalizations (USVs) in newborns, pups aged from 2 days (P2) up to 13 days (P13) underwent a 5-minute maternal separation test (Fig. 1B), during which USVs were continuously recorded (Fig. 1C). In the control group (*n* = 30), the number of USVs produced during the test increased from 176.4 +/- 150.3 at P2-3 to 469.1 +/- 170.4 at P6-7 and then decreased to 155.1 +/- 180.7 at P12-13 (Fig. 1D). An alteration of this Gaussian-like pattern of ultrasonic vocalization development was observed in neonates from TiO2NPs-exposed litters (*n* = 24, Fig. 1D), particularly in females, in which the peak of the Gaussian curve was delayed by one day, shifting from P6-7 to P8-9 (Fig. 1E, F). In addition to this modification in the kinetics of USV development, both female and male TiO2NPs-exposed pups produced significantly fewer USVs during the maternal separation test at P6-7 compared to control animals (Females, P6-7: Sidak’s test, *p* = 0.0008; Males, P6-7: Sidak’s test, *p* = 0.0469; Fig. 1E, F). Next, we calculated the total time during which pups produced USVs during the 5-minute maternal separation test (Fig. 1G-I), and observed a similar trend over the P2-P13 period for all groups. This indicates that, overall, both female and male TiO2NPs-exposed pups spent less time vocalizing compared to non-exposed animals at P6-7, the time corresponding to the peak of USV production (Females, P6-7: Sidak’s test, *p* = 0.0003; Males, P6-7: Sidak’s test, *p* < 0.0020; Fig. 1H, I). Taken together these results show that neonatal exposure to TiO2NPS disrupts the normal developmental trajectory of ultrasonic vocalizations in pups. This disruption is characterized by a delayed peak in USV production and a significant reduction in the number and duration of vocalizations at P6-7.

Given the important role of USVs in early-life physiological regulation, the alterations in USV production described above may be associated with changes in other developmental parameters. Here, we show that the body weight of TiO2NPs-exposed pups is significantly lower than that of non-exposed animals, starting at P8 in females (2 Way ANOVA test, all *p* < 0.0001; Figure S1C) and at P6 in males (2 Way ANOVA test, all *p* < 0.0001; Figure S1D), resulting in a significantly reduced weight gain over the P0-P13 period in TiO2NPs-exposed pups (t-test, *p* < 0.0001; Figure S1E). In contrast, other developmental parameters, including age of eye opening and performance in the righting reflex test, were not affected in exposed pups (Figure S1F-H). Both TiO2NPs-exposed and non-exposed pups showed a progressive decrease in the time required to perform the righting reflex test, with no significant differences between the two groups at any age tested (2 Way ANOVA test, age factor: *p* < 0.0001; treatment factor: *p* = 0.4692; Figure S1F). Similarly, no differences were observed in the developmental progression of eye opening or ear opening between TiO2NPs-exposed and non-exposed pups. Eye opening began at P11, with all pups having opened their eyes by P14 (2 Way ANOVA test, age factor: *p* < 0.0001; treatment factor: *p* = 0.4571; Figure S1G), while ear opening began at P3, with all pups having opened their ears by P5 (2 Way ANOVA test, age factor: *p* < 0.0001; treatment factor: *p* > 0.9999; Figure S1H). Overall, these findings indicate that neonatal exposure to TiO2NPs selectively alters early communicative and growth-related developmental processes without broadly impairing sensorimotor maturation.

**Fig. 2:**
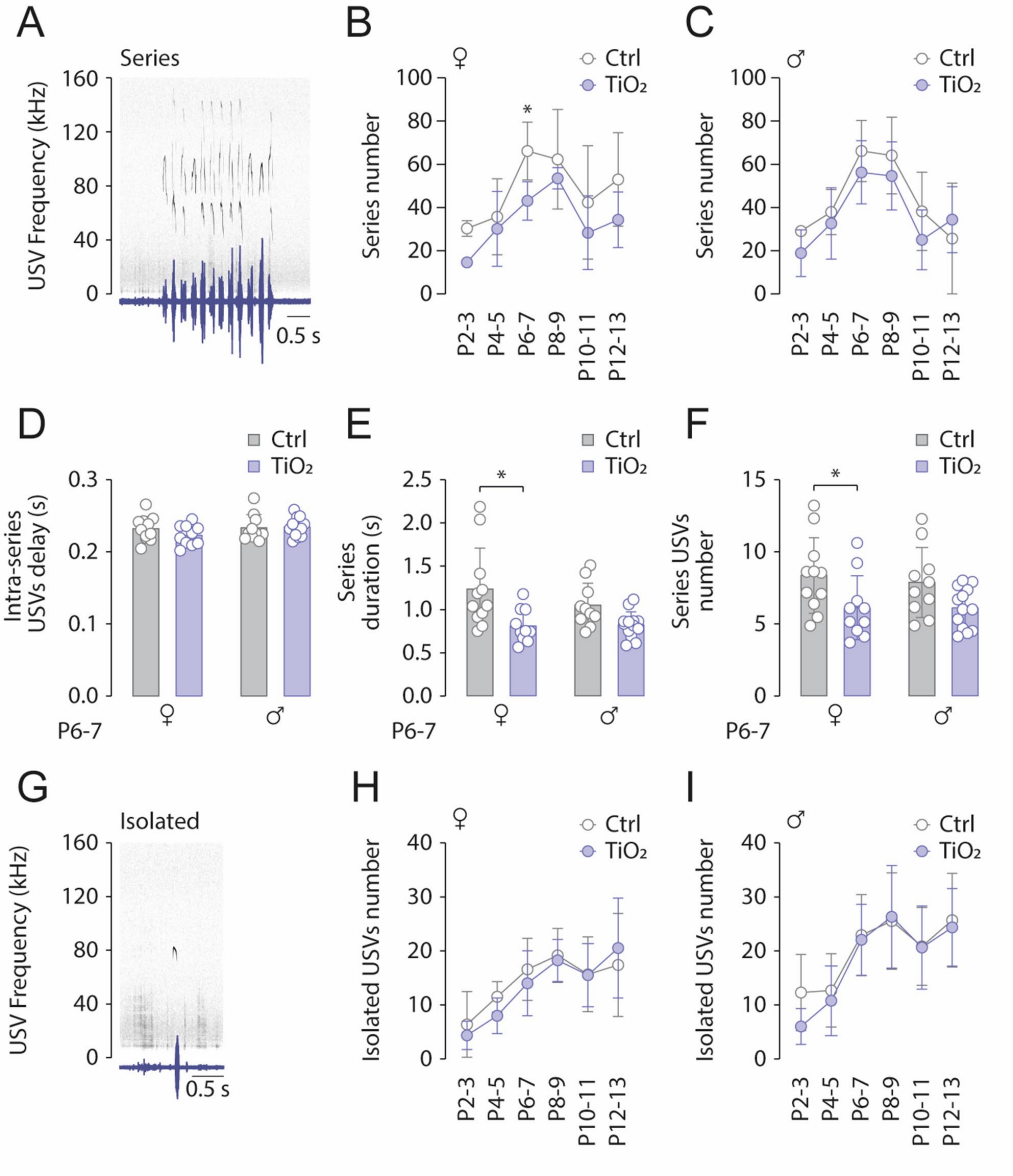
Perinatal exposure to TIO2NPs affects USV series production. **A** Sonogram illustrating a P7 control female pup producing a USV series. **B**,** C** Scatter plots comparing the number of USV series produced by control (light gray) and TiO2NPs-exposed (light blue) female (**B**) and male pups (**C**). **D–F** Bar charts representing intra-series interval (**D**), series duration (**E**), and the average number of USVs produced per series (**F**) in control (light gray bars) and TiO2NPs-exposed (light bluebars) P6-7 females and males. **G** Audio recording showing a P7 control female pup producing isolated USVs. **H–I** Scatter plots depicting the evolution of isolated USV counts over the P2-P13 period for control (light gray) and TiO2NPs-exposed (light blue) female (**H**) and male (**I**) pups. Number of female Control pups: 14; Number of male Control pups: 10; Number of female TiO2NPs pups: 12; Number of male TiO2NPs pups: 12

When examining the overall organization of vocalizations, we observed that USVs are produced either in grouped sequences, referred to as phrases, forming rhythmic series with repeated calls (Fig. 2A), or as isolated calls (Fig. 2G). Thus, we next assessed whether the reduction in USVs observed in TiO2NPs-exposed pups affects both isolated calls and USV series (Fig. 2). At P6-7 (Control: *n* = 24; TiO2NPs: *n* = 24) the total number of series produced during the 5-minutes maternal separation test was reduced in TiO2NPs-exposed females but not males (Females: Sidak’s test, *p* = 0.017; Males: Sidak’s test, *p* = 0.4025; Fig. 2B, C). This reduction was not accompanied by a decrease in the interval between two USVs within the same series (Females: Sidak’s test, *p* = 0.3439; Males: Sidak’s test, *p* = 0.9780; Fig. 2D), but can be linked to a decrease in both the duration of the series in Females (Females: Sidak’s test, *p* = 0.0042; Males: Sidak’s test, *p* = 0.1456; Fig. 2E), and the average number of USVs produced per series (Females: Sidak’s test, *p* = 0.0460; Males: Sidak’s test, *p* = 0.1214; Fig. 2F). On the other hand, the production of isolated USVs was not affected in either female or male TiO2NPs-exposed pups (all Sidak’s tests, *p* > 0.05; Fig. 2H, I). In all groups, isolated USVs production increased from P2-3 and reached a plateau at P8-13. In conclusion, our results indicate that the reduction in USV number at P6-7 is due to a decrease in series production rather than a change in the number of isolated USVs.

### Alteration of USV frequency parameters and USV types in exposed animals

**Fig. 3:**
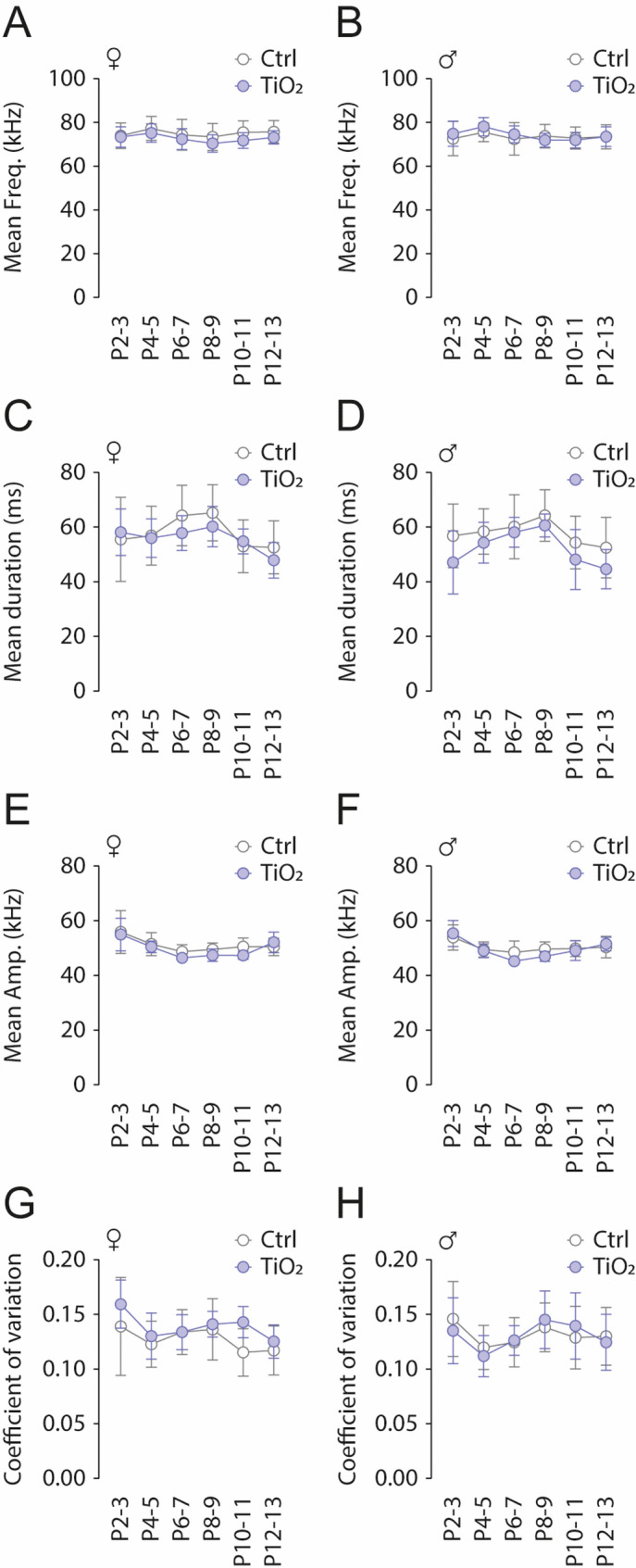
Perinatal exposure to TiO2NPs does not alter overall USV frequency parameters. **A**,** B** Scatter plots showing the evolution of mean USV frequency during the 5-minute maternal separation test across the P2-P13 period for control (light gray) and TiO2NPs-exposed (light blue) female (**A**) and male (**B**) pups. **C–H** Similar representations for mean USV duration (**C**,** D**), mean amplitude (**E**,** F**), and coefficient of variation (**G**,** H**). Number of female Control pups: 14; Number of male Control pups: 10; Number of female TiO2NPs pups: 12; Number of male TiO2NPs pups: 12

**Fig. 4:**
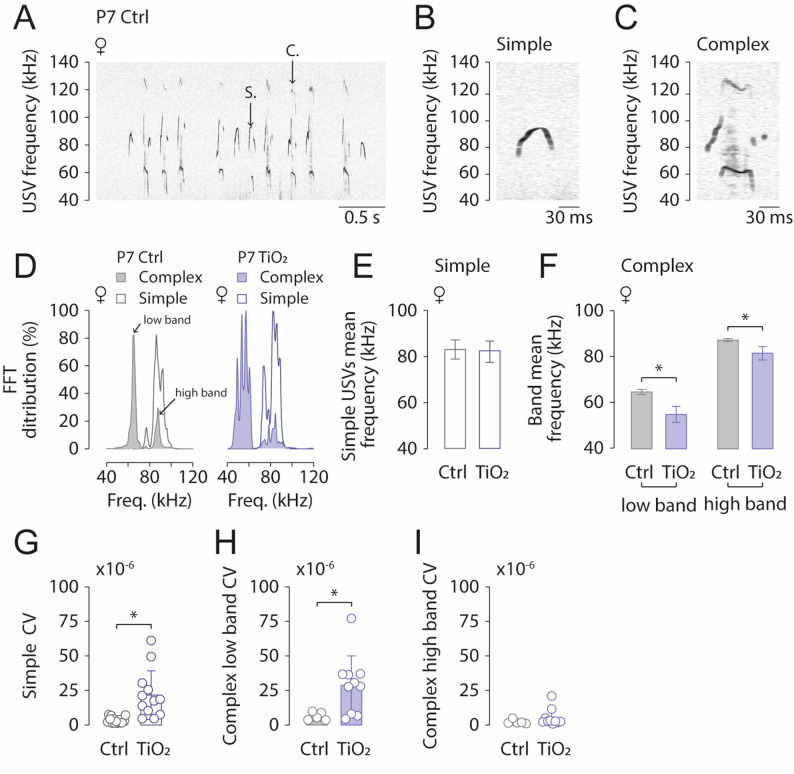
Perinatal exposure to TiO2NPs affects USV frequency bands. (**A**) Sonogram of a P7 control female pup producing Simple (S.) and Complex (C.) USVs. (**B**,** C**) Enlarged sections from (A), highlighting a Simple (S.) and a Complex (C.) USV. (**D**) Fast Fourier Transform (FFT) analysis illustrating individual frequency components of Simple and Complex USVs in control (light gray) and TiO2NPs-exposed (light blue) pups. (**E**,** F**) Bar charts comparing the mean frequency of Simple (**E**) and Complex (**F**) USVs between control and TiO2NPs-exposed pups. (**G-I**) Similar representation for the coefficient of variation of Simple (**G**) and Complex (**H**,** I**) USVs. Number of Control pups: 12; Number of TiO2NPs pups: 12

A key aspect of USV-based communication is the frequency range utilized, as it plays a crucial role in the mother’s ability to interpret the signals accurately. Here, we report no differences between exposed (*n* = 24) and non-exposed pups (*n* = 24) in USV frequency-related parameters. Specifically, there were no significant effect on USV mean frequency (Females: 2 Way ANOVA test, all *p* > 0.05; Males: 2 Way ANOVA test, all *p* > 0.05; Fig. 3A, B), USV mean duration (Females: 2 Way ANOVA test, all *p* > 0.05; Males: 2 Way ANOVA test, all *p* > 0.05; Fig. 3C, D), USV mean amplitude (Females: 2 Way ANOVA test, all *p* > 0.05; Males: 2 Way ANOVA test, all *p* > 0.05; Fig. 3E, F) or USV coefficient of variation (Females: 2 Way ANOVA test, all *p* > 0.05; Males: 2 Way ANOVA test, all *p* > 0.05; Fig. 3G, H). We observed that pups produced two main types of USVs: simple and complex (Fig. 4A-C). Both types were observed either as isolated USVs or within USV series. Using a Fast Fourier Transform (FFT) we extracted the spectral components of simple and complex USVs of P6-7 pups (Fig. 4D). Frequencies of simple USVs followed a Gaussian-like distribution, with a mean frequency of 83.7 ± 5.0 kHz (Fig. 4E). In contrast, the frequency distribution of complex USVs exhibited two main peaks at 64.6 ± 1.0 kHz (low band) and 81.4 ± 2.9 kHz (high band) (Fig. 4F). Exposed pups also produced both simple and complex USVs (Fig. 4D, right). When analyzing temporal parameters, we found no difference in the mean frequency of simple USVs between TiO2NPs-exposed (*n* = 12) and control pups (*n* = 12), (t-test, *p* = 0.5722; Fig. 4E). However, TiO2NPs-exposed pups produced complex USVs with significantly lower mean frequencies in both the low and high bands compared to controls (low band: t-test, *p* < 0.0001; high band: t-test, *p* = 0.0006; Fig. 4F). To assess the spread of frequency distribution, we calculated the coefficient of variation (CV) for each frequency band (Fig. 4G). TiO2NPs-exposed pups exhibited significantly higher CV values in simple USVs and the complex low band, but not in the complex high band, compared to controls (simple USVs CV: t-test, *p* = 0.0003; low band CV: t-test, *p* = 0.0341; high band CV: t-test, *p* = 0.2962; Fig. 4G-I). Taken together, our findings suggest that while overall USV frequency-related parameters remain unchanged in TiO2NPs-exposed pups, the variability in frequency distribution is increased.

### Perinatal exposure to TIO2NPs alters USV syntax

**Fig. 5:**
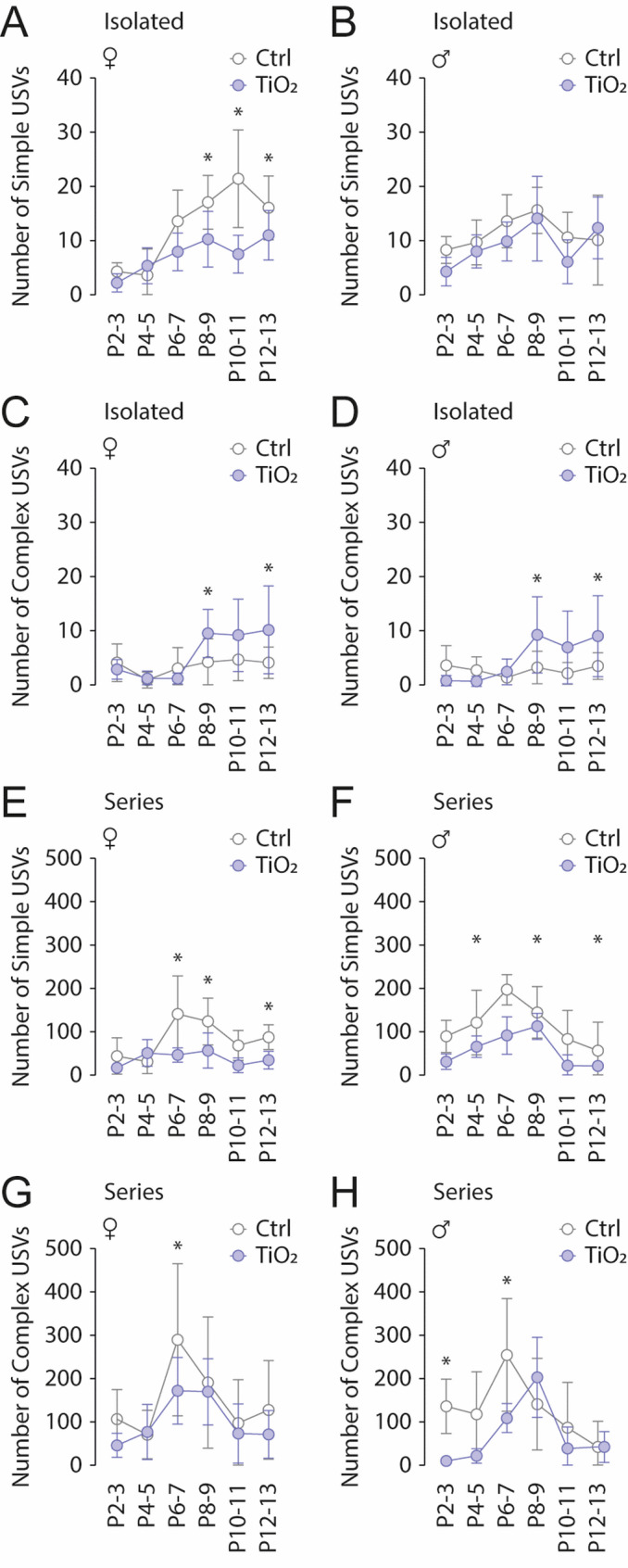
Perinatal exposure to TiO2NPs alters the number of Simple and Complex USV produced over the P2-P13 period. **A**,** B** Scatter plots showing changes in simple USV number in isolated USVs over the P2-13 period in Control (light grey bars) and TiO2NPs-exposed (light blue bars) female (**A**) and male (**B**) pups. **C**,** D** Similar representation for complex USV number in isolated USVs. **E**,** F** Similar representation for simple USV number in series of USVs. **G**,** H** Similar representation for complex USV number in series of USVs. Number of female Control pups: 12; Number of male Control pups: 10; Number of female TiO2NPs pups: 12; Number of male TiO2NPs pups: 12

We recorded both simple and complex USVs from postnatal day 2 to 13, occurring either in isolation or as part of a series (Fig. 5). Analysis of isolated USVs revealed that exposed female pups (*n* = 12) emitted fewer simple USVs compared to non-exposed females (*n* = 12). This difference was not observed in males (control: *n* = 10; exposed: *n* = 12), for which the number of simple USVs remained comparable between groups across all developmental stages (Females: Sidak’s test, P8-9, P10-11, and P12-13: *p* < 0.05; Males: Sidak’s test, all *p* > 0.05; Fig. 5A, B). In contrast, while complex USV production remained stable in control animals throughout the P2-P13 period, exposed pups exhibited a significant increase in complex USV output during the P8-P13 window (Females: Sidak’s test, P8-9 and P12-13: *p* < 0.05; Males: Sidak’s test, P8-9 and P12-13: *p* < 0.05; Fig. 5C, D). In series, both exposed females and males produced fewer simple USVs compared to non-exposed pups across the P2-P13 period (Females: Sidak’s test, P6-7, P8-9, and P12-13: *p* < 0.05; Males: Sidak’s test, P2-3, P6-7, and P10-11: *p* < 0.05; Fig. 5E, F). A reduction in complex USVs within series was also observed at P6-7 in both sexes (Females: Sidak’s test, P6-7: *p* < 0.05; Males: Sidak’s test, P2-3 and P6-7: *p* < 0.05; Fig. 5G, H). Overall, these findings indicate that exposure to TiO2NPs leads to a general reduction in simple USV production from P2 to P13, with a pronounced decrease within series in both simple and complex USVs at P6-7.

**Fig. 6:**
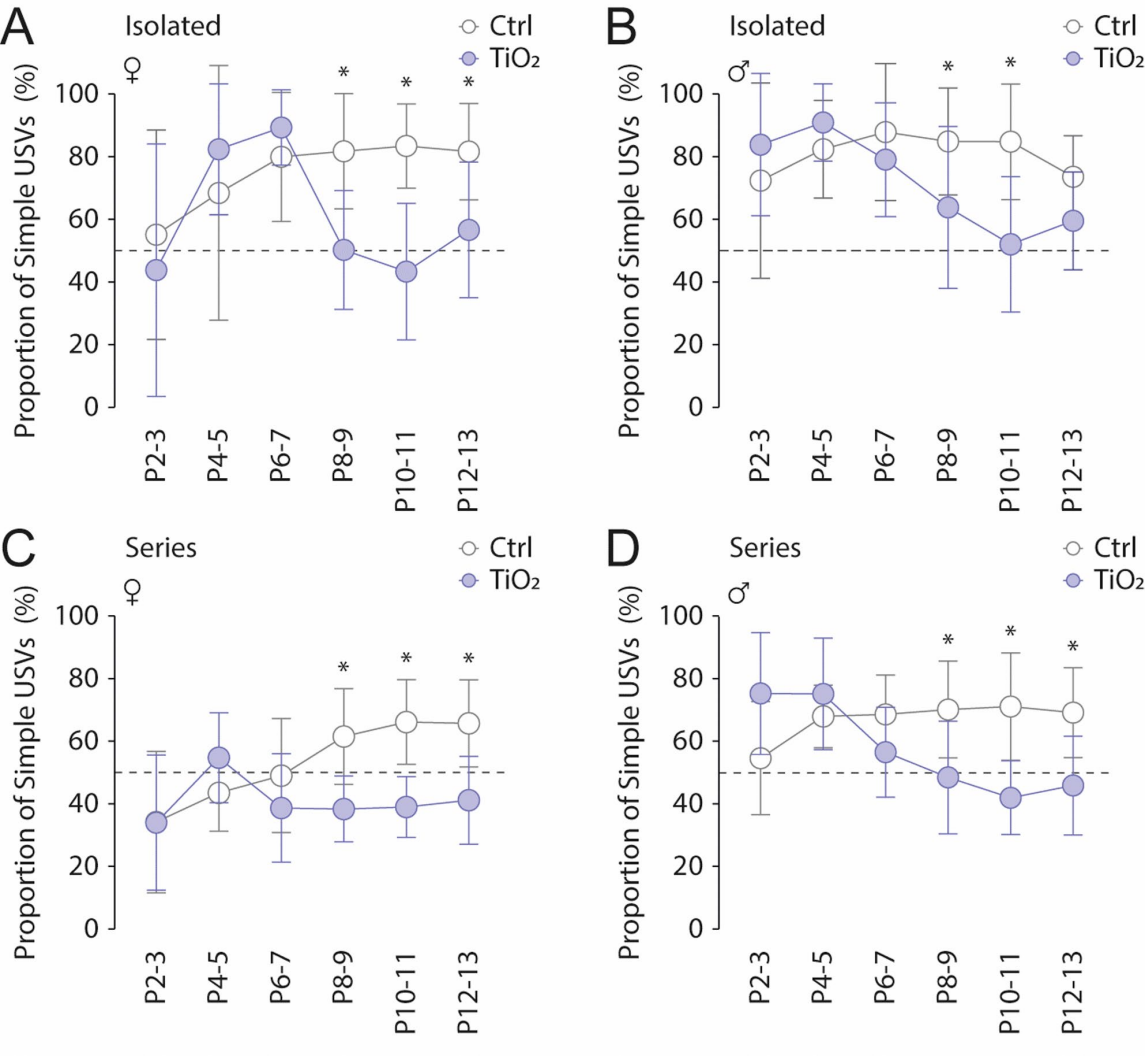
Perinatal exposure to TiO2NPs alters the Simple-to-Complex USV ratio over the P2-P13 period. **A**,** B** Scatter plots showing changes in the Simple-to-Complex USV ratio for isolated USVs over the P2-13 period in Control (light grey bars) and TiO2NPs-exposed (light blue bars) female (**A**) and male (**B**) pups. **C**,** D** Similar representation for USV series. Number of female Control pups: 12; Number of male Control pups: 10; Number of female TiO2NPs pups: 12; Number of male TiO2NPs pups: 12

In mice, the prevalence of specific USVs changes progressively throughout development [4, 26]. Thus, we analyzed the evolution of the proportion of USV produced over the P2-P13 period in the maternal separation test (Fig. 6). At P2-3, control pups (*n* = 22) produced a proportion of simple to complex isolated USVs of 56.8 +/- 29.3% and 73.2 +/- 31.4% in females and males, respectively (Fig. 6A, B). Over the following days, the proportion of simple to complex isolated USVs increased, reaching 84.3 ± 13.5% in females at P10-11 and 88.9 ± 22.0% in males at P6-7. In contrast, TIO2NPs-exposed pups (*n* = 24) produced relatively fewer simple isolated USVs during the same developmental period (Females: Two-way ANOVA, treatment factor: *p* = 0.0032; Males: Two-way ANOVA, treatment factor: *p* = 0.0291; Fig. 6A, B), as their proportion of simple to complex isolated USVs tended toward 50% in both females and males throughout the P6-P13 period (Fig. 6A, B). Simple and complex USVs were also observed in series of USVs during the P2-P13 period both in females and males (Fig. 6C, D), and the proportion of simple USVs also increased over this developmental period in control animal (Females, P2-3: 33.6 +/- 22.7, P12-13: 65.4 +/- 14.0; Males, P2-3: 54.3 +/- 18.2, P12-13: 69.0 +/- 14.5), but not in TIO2NPs-exposed pups that produced significantly a lower proportion of simple USVs (Females: Two-way ANOVA, treatment factor: *p* = 0.0017; Males: Two-way ANOVA, treatment factor: *p* = 0.0191; Fig. 6C, D).

**Fig. 7:**
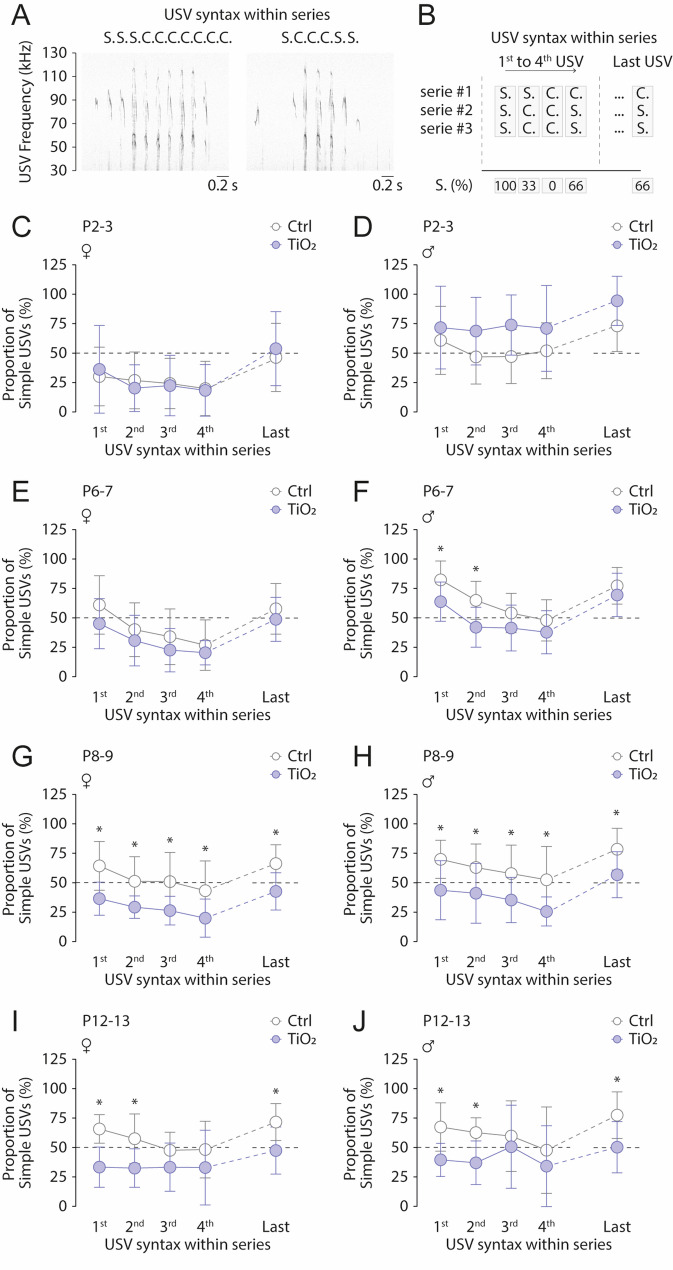
Perinatal exposure to TiO2NPs affects USV series syntax development over the P2-P13 period. **A** Sonograms illustrating USV syntax within series. **B** Schematic representation of the method used to calculate mean syntax from the first four and last USVs in a series. **C**,** D** Scatter plots showing mean syntax use in P2-3 female (**C**) and male (**D**) pups. **E-J** Similar representations for P6-7 (**E**,** F**), P8-9 (**G**,** H**), and P12-13 (**I**,** J**) pups. Number of female Control pups: 12; Number of male Control pups: 10; Number of female TiO2NPs pups: 12; Number of male TiO2NPs pups: 12

In mice, although the vocal repertoire remains stable during development, the composition and structure of phrases (i.e. syntax of series) undergo significant changes within the first few postnatal days [4]. Thus, we next assessed the possible alterations of USVs syntax development over the P2-P13 period, following TIO2NPs exposure (Fig. 7A). USVs were analyzed sequentially within series to calculate the simple-to-complex proportion for each consecutive USV, from the first to the fourth, as well as for the final USV in the series (Fig. 7B). We report a syntax alteration that progressively emerges during the first two postnatal weeks. In both females and males, this alteration is not visible at the early stages of development (P2-P7); it emerges at P8-9 and persists at least until P12-13 (Fig. 7). At P2-3, in both females and males, we observed no differences in series syntax between non-exposed (*n* = 22) and TiO2NPs-exposed (*n* = 24) animals (Females: 2 Way ANOVA test, all *p* > 0.05; Males: 2 Way ANOVA test, all *p* > 0.05; Fig. 7C, D). In non-exposed and TiO2NPs-exposed female pups, USV series were composed mostly of complex USVs, regardless of their position within the series (Fig. 7C), except for the last one, which had a similar probability of being either a simple or complex USV. In males at P2-3, the Simple-to-Complex USV proportion was higher in comparison to females, being either close to 50% in non-exposed animals and above 50% in TiO2NPs-exposed animals (Fig. 7D). At P4-5, the syntax was similar for all groups when compared to P2-3 (Figure S2). Starting at P6-7, a new syntactic pattern emerged in non-exposed animals (Figure S2, Fig. 7E-J). Briefly, within the series, the first USV was predominantly a simple USV, with a proportion of occurrence of approximately 66%. This proportion gradually declined in the subsequent USVs before rising again in the final one, forming a U-shaped pattern. This maturation of USV series syntax was however not observed in both females and males TiO2NPs-exposed pups, not even at the most advanced stages (P12-13) (Fig. 7E-J, Figure S2). Together, these findings demonstrate that maternal TiO2NPs exposure significantly alter series structure and call proportions in pups. These results suggest that maternal TiO2NPs exposure disrupts normal vocal production mechanisms, including those influencing the structure of USV series.

### Activity of the intermediate reticular oscillator is depressed in exposed pups

**Fig. 8:**
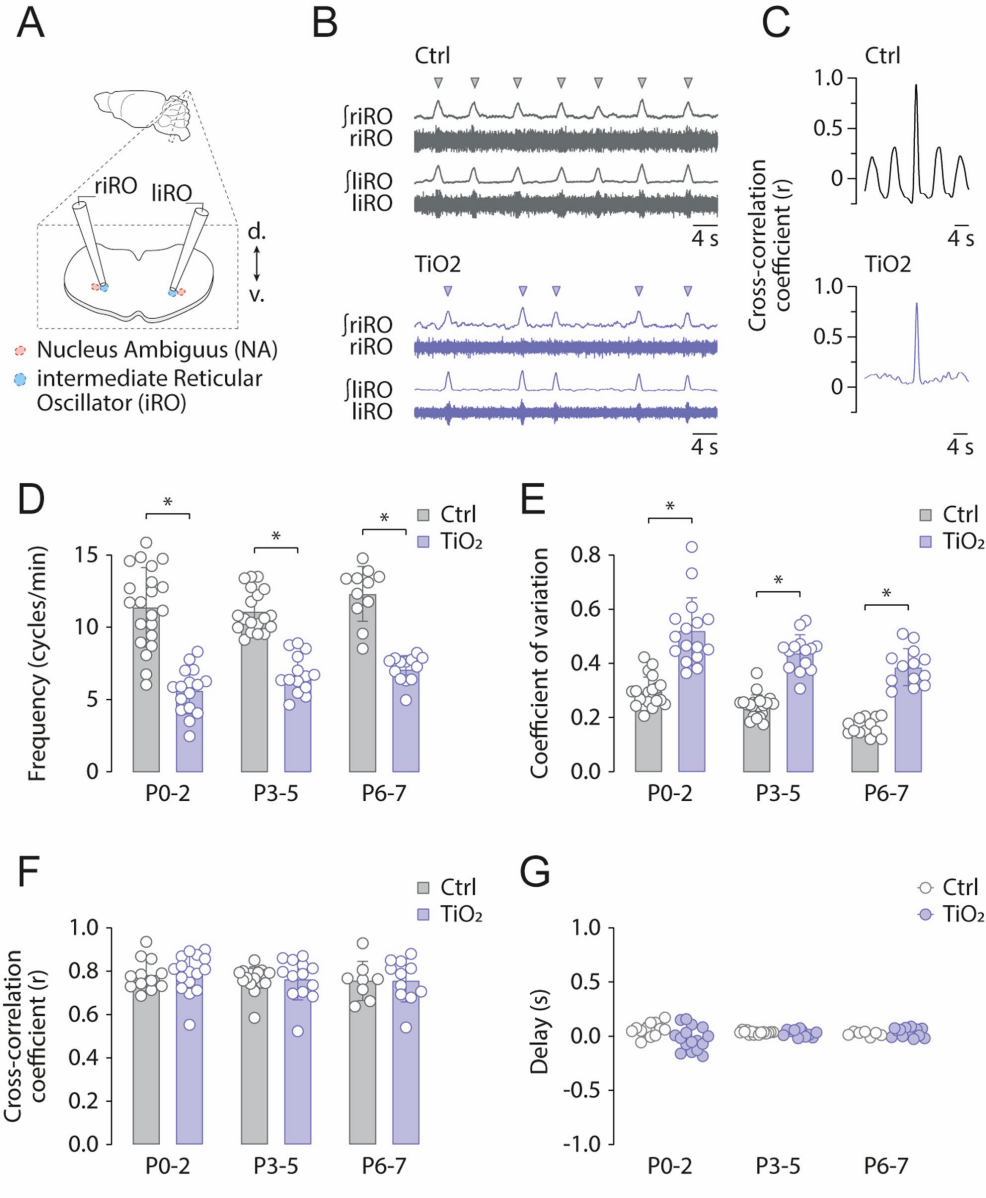
Perinatal exposure to TiO2NPs disrupts the intermediate reticular oscillator (iRO) activity. **A** Schematic representation of the experimental protocol. **B** Bilateral electrophysiological recordings of the intermediate Reticular Oscillator (iRO) in control (light gray traces) and TiO2NPs-exposed (light blue traces) pups. Triangles indicate bursts of action potentials. **C** Cross-correlation coefficients of contralateral iRO activities in control and TiO2NPs-exposed pups. **D** Bar chart showing the mean frequency of iRO bursts of action potentials over the P0-7 period. **E–G** Similar representations for the coefficient of variation (**E**), cross-correlation coefficient (**F**), and mean delay between contralateral iRO activities (**G**). Number of Control pups: P0-2: 21; P3-5: 22; P6-7: 13; Number of TiO2NPs pups: P0-2: 16; P3-5: 14; P6-7: 13;

Recent studies have identified the intermediate Reticular Oscillator (iRO) as a brainstem neural center involved in USV production [20–22]. To determine whether the alterations we observed in USV production were linked to changes in iRO activity, we conducted electrophysiological recordings on isolated ex-vivo brainstem slices containing the iRO from P0 to P7 aged pups (control: *n* = 56; TiO2NPs: *n* = 43; Fig. 8A). At P0-2, both left and right iROs from control animals were active rhythmically and produced rhythmical bursts of action potentials at a frequency of 11.3 +/- 2.7 cycles per minute (Fig. 8B, D). In comparison, the iRO from TiO2NPs-exposed pups of the same age generated bursts of action potentials at a lower frequency of 5.5 ± 1.5 cycles per minute, indicating reduced excitability of this oscillator (Sidak’s test, *p* < 0.0001; Fig. 8D). Next, to evaluate the stability of iRO oscillations we calculated the coefficient of variation of the frequency of iRO bursts of action potentials. This parameter was significantly higher in TiO2NPs-exposed pups, revealing a lower stability of iRO oscillators in exposed pups (Sidak’s test, *p* < 0.0001; Fig. 8E).

The production of USV requires a tight synchronization of contralateral muscles, ensuring precise control of airflow and sound frequency for optimal vocalization. Using the same approach as described above, we show that at P0-2, the bilateral synchronization required for USV production is achieved directly at the brainstem level. In control animals, bilateral iROs exhibit a high degree of coupling (mean cross-correlation coefficient: 0.77 ± 0.07; Fig. 8C, F). Similarly, TiO2NPs-exposed pups also showed a high degree of coupling between contralateral iROs (mean cross-correlation coefficient: 0.78 ± 0.08), with no significant difference compared to control pups (2 Way ANOVA test, all *p* > 0.05; Fig. 8C, F). Additionally, left and right iRO activities showed minimal delays in both control and TiO2NPs-exposed P0-2 pups (delay: control, 0.05 +/- 0.05 s; TiO2NPs-exposed, -0.01 +/- 0.09 s; 2 Way ANOVA test, all *p* > 0.05; Fig. 8G).

Next, we sought to determine whether the functional alterations of iRO observed at birth in exposed animals persisted in the following days. Notably, compared to P0-2 pups, similar observations were made at later developmental stages (P3-5 and P6-7). Specifically, in TiO2NPs-exposed pups, iRO frequency was significantly reduced compared to control pups at both P3-5 (Sidak’s test, *p* < 0.0001) and P6-7 (Sidak’s test, *p* < 0.0001; Fig. 8D). Additionally, the coefficient of variation was significantly increased at both stages (P3-5: Sidak’s test, *p* < 0.0001; P6-7: Sidak’s test, *p* < 0.0001; Fig. 8E). However, no significant differences were observed in the cross-correlation coefficient (two-way ANOVA, all *p* > 0.05; Fig. 8C, F) or bilateral delay (two-way ANOVA, all *p* > 0.05; Fig. 8G).

These findings indicate that in TiO2NPs-exposed pups, iROs exhibit reduced excitability and increased activity variability but no alteration in bilateral coupling, at least during the first week of life. The persistence of these effects suggests a sustained impact on iRO development, potentially affecting maternal-infant interactions during this critical period.

### USV from exposed pups fail to elicit appropriate maternal guidance

**Fig. 9:**
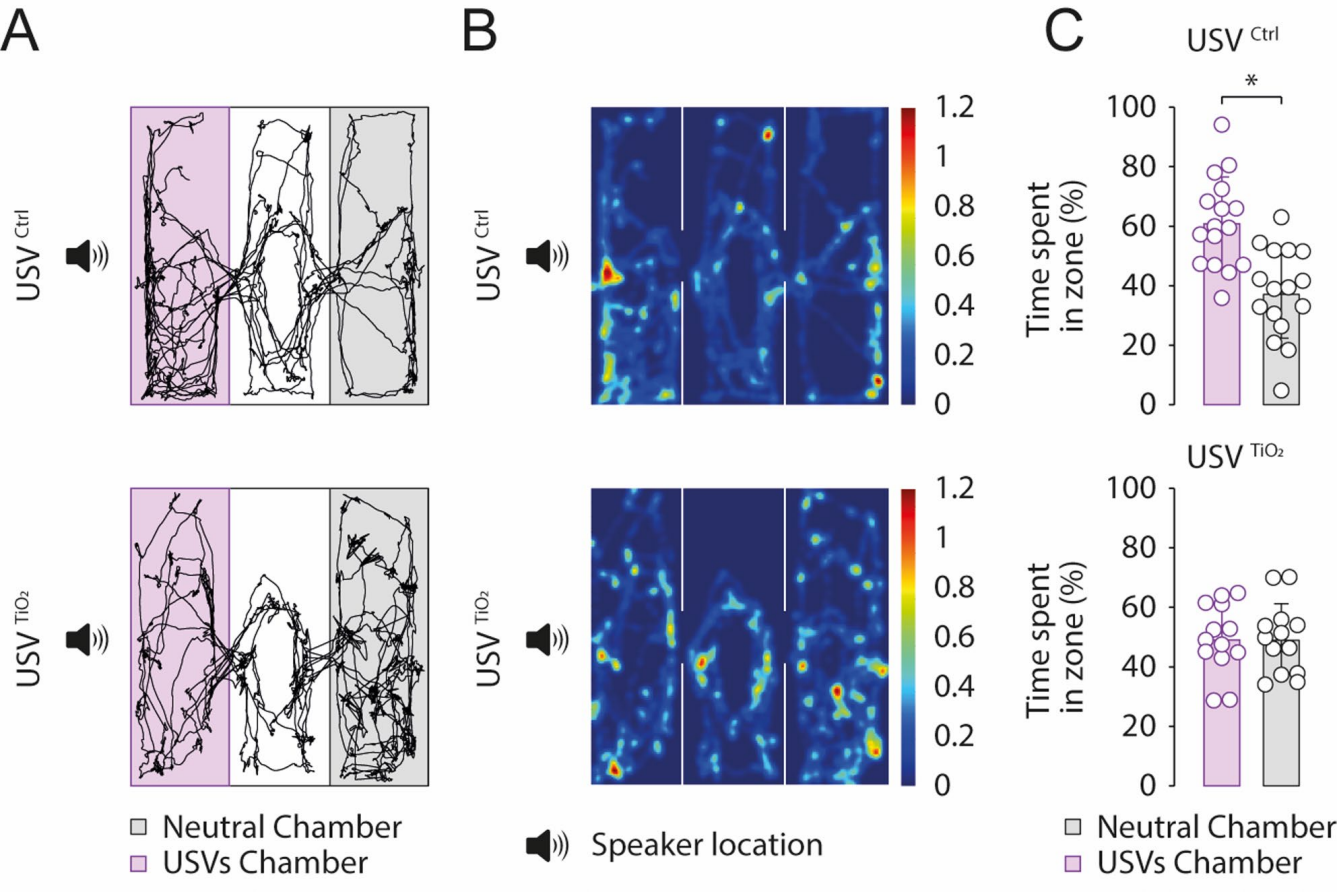
USVs from TiO2NPs-exposed pups fail to elicit maternal guidance in non-exposed dams. **A** Schematic representation of the playback experiment protocol. The USV chamber (speaker location) is shaded in light pink, and the neutral chamber is in light gray. Black traces indicate the movement of a non-exposed adult female when USVs from control (upper) or TiO2NPS-exposed pups (middle) were played. **B** Heat map highlighting preferred stopping zones (in red) of adult females during the test. **C** Bar charts showing the time spent by adult females in the USV chamber (light pink bars) vs. the neutral zone (light gray bars) when presented with USVs from control (USV^CTRL^) or TiO2NPs-exposed (USV^TiO2^) pups. Number of females: USV^CTRL^: 21; USV^TiO2^: 16

The emission of USVs by pups is essential for triggering appropriate maternal responses during a developmental period when pups have the motor ability to leave the maternal nest but lack the sensory capabilities to return or survive outside of it. In the next series of experiments, we conducted playback experiments using a three-chamber test to assess the ability of USVs emitted by either control or TiO2NPs-exposed pups to elicit maternal attraction toward the sound source (Fig. 9A). When USV emitted by control pups (USV^CTRL^) was played, females (*n* = 21) spent significantly more time in the USV chamber compared to the neutral chamber (no sound) (t-test, t = 4.362, *p* < 0.0001; Fig. 9B, C). In contrast, no chamber preference was observed in females (*n* = 16) for which playback USVs originated from TiO2NPs-exposed pups (USV^TiO2^) (t-test, t = 0.0323, *p* = 0.9741; Fig. 9B, C). These results are not due to a specific effect of TiO2NPs exposure on dams, as similar results were observed in TiO2NPs-exposed females (Figure S3). Indeed, these females spent significantly more time in the USV chamber than in the neutral chamber when USV emitted by control pups (USV^CTRL^) was played (t-test, t = 3.637, *p* = 0.0019; Figure S3A-C, top), whereas no chamber preference was observed during playback of USVs emitted by TiO2NPs-exposed pups (USV^TiO2^) (t-test, t = 0.2176, *p* = 0.8302; Figure S3A-C, bottom). Our results show that while USVs from control pups effectively triggered maternal attraction, those from TiO2NPs-exposed pups failed to do so, suggesting that TiO2 exposure may alter the communicative effectiveness of USVs. These results emphasize the potential impact of early-life environmental factors on neural mechanisms underlying vocal communication and maternal-infant interactions.

**Fig. 10:**
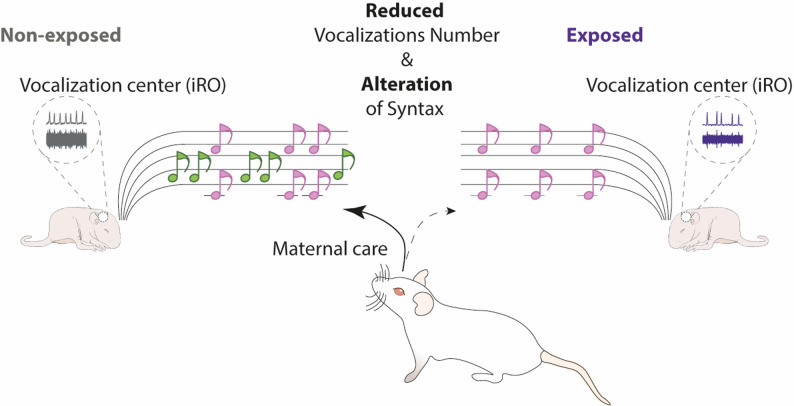
Schematic representation of the consequences of perinatal TiO2NPs exposure on USV production in mouse offspring

## Discussion

### Mechanisms of TiO2 nanoparticle action on the developing brain

It is well established that perinatal exposure to a wide range of substances can alter brain development and, consequently, the production of USVs in newborns. Substances such as dipyrone [27], 2,3,7,8-tetrabromodibenzofuran [28], valproic acid [29, 30], cannabidiol [30, 31], oxycodone [32], and neonatal opioid withdrawal [33] have all been shown to impact USV production. Additionally, exposure to environmental pollutants such as 2-chloro-3,7,8-tribromodibenzofuran has also been reported to disrupt USV production in newborn mice [34]. Finally, a single intravenous administration of a high dose (1 mg) of TiO2NPs to pregnant dams on gestational day 9 has been shown to reduce USV production in their offspring [35]. Thus, alterations in USV production can probably not be attributed to the disruption of a single cellular pathway or mechanism. Rather, they likely reflect more widespread disturbances in central nervous system development, which represent one of the many consequences of TiO2NPs exposure [36]. Although recent studies have shown that TiO2NPs can induce mitochondrial damage in neuroblastoma cells [37], they have also been reported to interfere with critical developmental processes such as neurogenesis, cell proliferation, and apoptosis, potentially leading to long-term impairments in CNS development and function [38–41]. Importantly, TiO2NPs bind to cellular prion protein (PrPC) disrupting its normal function and leading to increased production of reactive oxygen species, as well as the accumulation of tumor necrosis factor receptors (TNFR) on the surface of brain neurons [16]. These cellular disturbances resulting from PrPC dysfunction are associated with a wide range of functional impairments, including anxiety [42–45], deficits in social recognition [46], and memory impairments [47, 48].

Currently, while no clear link can be established at the cellular or subcellular level between nanoparticle exposure and vocalization deficits, evidence may emerge at the organismal level. Our results indicate that exposure to TiO2NPs does not alter classical developmental milestones commonly used to assess neonatal impairment, such as age of eye opening and performance in the righting reflex test. In contrast, impaired growth may be associated with altered ultrasonic vocalization (USV) production. In support of this, we show that perinatal exposure to TiO2NPs leads to reduce weight gain in pups during the postnatal period [19]. This reduced growth may be linked to several factors. First, a decrease in USV production may alter maternal feeding behavior toward exposed pups. Second, TiO2NPs accumulation in the mammary gland, causing tissue damage, oxidative stress, and disruption of the blood-milk barrier [15, 49, 50]. Similar developmental patterns have been observed in other models, such as Ts65Dn mice, where USV deficits are accompanied by delayed developmental milestones such as a reduced body weight gain [51]. Additionally, Wingfield et al. (2025) reported increased USV emissions and altered syllable patterns at P14, alongside a reduced body weight from P1 to P14 during neonatal opioid withdrawal [33]. These findings suggest a broader link between early-life growth deficits and altered communication behaviors, reinforcing the need to investigate how systemic developmental disruptions contribute to USV alterations and vice versa. In the long term, neonatal USV alterations may influence or predict individual adult behavior, including anxiety profiles [52] and sociability [53].

### TiO2 nanoparticle exposure alters USVs across multiple dimensions, including frequency and call types

Taken together, our results indicate that at least four parameters need to be considered: the decreased number of USVs at P6-P7, the delayed peak in the Gaussian-like developmental curve of USV production, the reduced low- and high-band mean frequencies in complex calls, and the altered call syntax within series all point to subtle disruptions in vocal control, which may impair pup-mother communication and, consequently, early neurodevelopment. First, reductions in the number of USVs or shifts in the developmental trajectory, for example, a blunted or delayed peak in the typical “Gaussian-like” curve of USV production, have been documented. In a prenatal Methylazoxymethanol acetate (MAM) rat model, pups failed to show the normal postnatal increase in call rate and emitted significantly fewer USVs by PND 12 compared to controls, with disrupted maternal-potentiation responses (i.e. weakened pup-mother communication) and lower call complexity [54]. Similarly, following early-life stress via repeated maternal separation, rat pups emitted fewer calls, or showed altered temporal patterns, suggesting that deviations in call number or developmental dynamics may signal early communication deficits [55]. Importantly, and consistent with our findings, alterations in USV number can be restricted to discrete developmental windows, as illustrated in genetic models of autism spectrum disorder such as Nf1 and Tsc2 mice, which exhibit reduced call production specifically at P6-P8 and P10, respectively [56]. This suggests that even a transient disruption of vocal output may reveal markedly abnormal maturation of underlying neural circuits.

Second, several studies report modifications in the acoustic properties of neonatal USVs, notably spectral features such as frequency, bandwidth, duration. For instance, in a genetic model of Fragile X syndrome, pups did not show fewer isolation-induced USVs, but their calls exhibited altered acoustic signatures: flat calls had a higher carrier frequency, complex calls had a wider frequency range, and the proportion of downward calls was reduced compared to wild-type littermates [57]. In environmental and genetic models of autism spectrum disorder (ASD), such as exposure to the pesticide Chlorpyrifos (CPF) or heterozygosity for Mthfr, pups showed significant alterations in start and end frequencies, call duration, and bandwidth of USVs. These spectral-temporal USV alterations are interpreted as early signs of impaired vocal regulation associated with neurodevelopmental risk factors [6].

Third, disruptions in USV syntax and temporal organization, defined as changes in syllable composition, sequence structure, transition probabilities between syllables, inter-call intervals, or call clustering, have been described in multiple neurodevelopmental models. In an in utero Valproic acid (VPA) mouse model of ASD, pups displayed not only reduced call rates and altered acoustic features, but also a degradation of higher-order USV sequence structure: mutual-information analyses revealed weaker dependencies between syllables, indicating loss of the complex syllable order typical of control pups [58]. In addition, the 2025 study on a double transgenic model (heterozygous Nf1 or Tsc2 mice) reported sex- and genotype-dependent alterations in the proportion of multisyllabic or “stacked” calls over the first postnatal days, evidence that genetic perturbations can specifically alter the composition and structure of the neonatal vocal repertoire [56]. Together, these data underscore that neonatal USV analysis should not be limited to call counting: combining measures of call number and developmental trajectory, acoustic quality, and syntax/temporal organization yields a more nuanced and sensitive assessment of early disruptions in communication. Such multi-dimensional USV impairments may serve as early behavioral biomarkers of disturbed neurodevelopment, with potential implications for later social, cognitive, or neural deficits.

### Limited contribution of maternal dysfunction to offspring vocal and developmental deficits

To determine whether the offspring phenotype primarily reflects maternal dysfunction or pup-intrinsic alterations, we assessed maternal responsiveness and developmental outcomes using playback-based and milestone analyses. Our playback experiments argue against a major contribution of maternal dysfunction to the phenotype observed in offspring. TiO2NPs-exposed dams displayed normal attraction toward ultrasonic vocalizations (USVs) emitted by control pups, while failing to respond to USVs produced by exposed pups. This dissociation indicates that maternal auditory processing and motivational responses remain largely preserved following TiO2NPs exposure, and that the impaired maternal attraction primarily reflects qualitative alterations in pup vocal signals rather than deficits in maternal sensory or motivational systems. Consistent with this interpretation, developmental alterations in exposed pups were selective rather than global. Although TiO2NPs-exposed pups exhibited reduced postnatal weight gain and marked abnormalities in ultrasonic vocalization production, other classical developmental milestones, including eye opening, ear opening, and righting reflex performance, were unaffected. Such a dissociated phenotype is inconsistent with a generalized developmental delay secondary to maternal respiratory impairment, chronic hypoxia, or global maternal care deficits, which typically result in broad delays across multiple sensorimotor and reflexive domains [59–61]. Taken together, while subtle maternal physiological or respiratory alterations cannot be entirely excluded, the converging behavioral, developmental, and playback evidence supports the conclusion that the vocal and developmental deficits observed in pups are predominantly driven by direct effects of perinatal TiO2NPs exposure on offspring neural and respiratory vocal circuits.

### Potential contribution of respiratory dysfunction to neonatal usv and developmental deficits

In the present study, we observed a marked reduction in ultrasonic vocalizations (USVs) during the maternal separation test. This finding aligns with our previous work showing that pups exposed to TiO2 nanoparticles exhibit reduced breathing frequency [19], confirming a causal link between respiratory function and vocal output. This raises an important question: could disruptions in respiratory control contribute not only to deficits in vocalization but also more broadly to impaired postnatal development? Indeed, prenatal hypoxia have been shown to impair sensorimotor maturation and reduce postnatal body weight, consistent with the developmental alterations we observed in our model [59]. Likewise, postnatal hypoxia induces increases in pro-inflammatory markers, oxidative stress, and ultimately abnormal maturation of neuronal circuits [60, 61]. However, these models typically expose animals to more severe hypoxic conditions that those experienced by pups exposed to TiO2 nanoparticles [19], even if a direct link between respiratory and vocalization deficits cannot be entirely ruled out, it is likely that distinct or additional mechanisms contribute to the developmental impairments observed here.

### Altered intermediate reticular oscillator function contributes to USV deficits

USV production is intrinsically coupled to respiratory activity, particularly through the intermediate reticular formation (iRO), as calls are generated specifically during expiration [20–22]. Disruptions in the development of the neural circuits controlling respiration can therefore simultaneously compromise both breathing and vocalization [62]. Furthermore, the instantaneous frequency of USVs during a call can correlate either positively or negatively with expiratory airflow, reflecting the tight interplay between vocal output and respiratory mechanics [21]. The iRO has been identified as a key hub in this process, with stimulation selectively initiating USV subtypes that are positively associated with expiratory airflow. In this context, the alterations in iRO activity observed in our study may underlie both the reduced number of USVs and the shifts in the Simple-to-Complex USV ratio reported in newborns. Importantly, these findings indicate that the observed deficits in USV production following TiO2 nanoparticle exposure are not simply secondary to general respiratory dysfunction, but likely reflect direct perturbations of vocal-respiratory integrative centers, with potential consequences for broader aspects of postnatal development.

### Disruption of neuromodulatory pathways may underlie impaired vocal-maternal interactions

Moreover, neuromodulatory systems appear to be particularly vulnerable to TiO2NPs. Previous studies have shown that acute exposure to TiO2NPs reduces the levels of critical neuromodulators such as serotonin, dopamine, and norepinephrine in the brain [14, 63]. Given the known involvement of these monoamines in regulating both affective behavior and vocalization [64], their disruption could partly underlie the behavioral deficits observed. This is supported by a study where pups lacking the main enzyme involved in serotonin production exhibit significantly reduced USV number [65]. Similar results were observed in genetic studies using 5HT receptor knockout models in neonatal isolation paradigms [66]. Of importance alteration of dopaminergic signaling in D2 receptor knockout mice significantly reduces USV production [67]. These studies underscore the critical role of both dopaminergic and serotonergic tone in early-life USV production and suggest that TiO2NPs may disrupt neonatal vocal behavior through combined effects on brainstem network function and neuromodulatory imbalance, warranting deeper investigation into how environmental pollutants impact neurodevelopment at multiple levels.

## Conclusion

Our findings indicate that perinatal exposure to TiO2NPs disrupts neonatal USV patterns. This disruption is characterized by a decreased number of calls, altered frequency band usage, impaired development of USV series syntax, and consequently, reduced ability to elicit maternal responses (Fig. 10). While the specific targets of TiO2NPs are yet to be fully identified, our results suggest that such exposure affects the excitability of the iRO, a key structure in USV generation. These findings highlight the potential for nanoparticle exposure during critical developmental windows to interfere with neural mechanisms underlying early communication, raising concerns about long-term neurodevelopmental consequences in offspring exposed to compromised maternal environments.

## Supplementary Information

Below is the link to the electronic supplementary material.

Supplementary Material 1.

## Data Availability

The datasets used and/or analyzed during the current study are available from the corresponding author on reasonable request.
