# Supplementary material for: Maternal exposure to titanium dioxide nanoparticles disrupts ultrasonic vocalization development in mouse offspring
Source: Part Fibre Toxicol. 2026 Apr 2;23:21. doi: 10.1186/s12989-026-00668-7 (PMC13104461; doi:10.1186/s12989-026-00668-7)
Supplement: Supplementary file 1 — Supplementary Material 1. [file 12989_2026_668_MOESM1_ESM.docx]

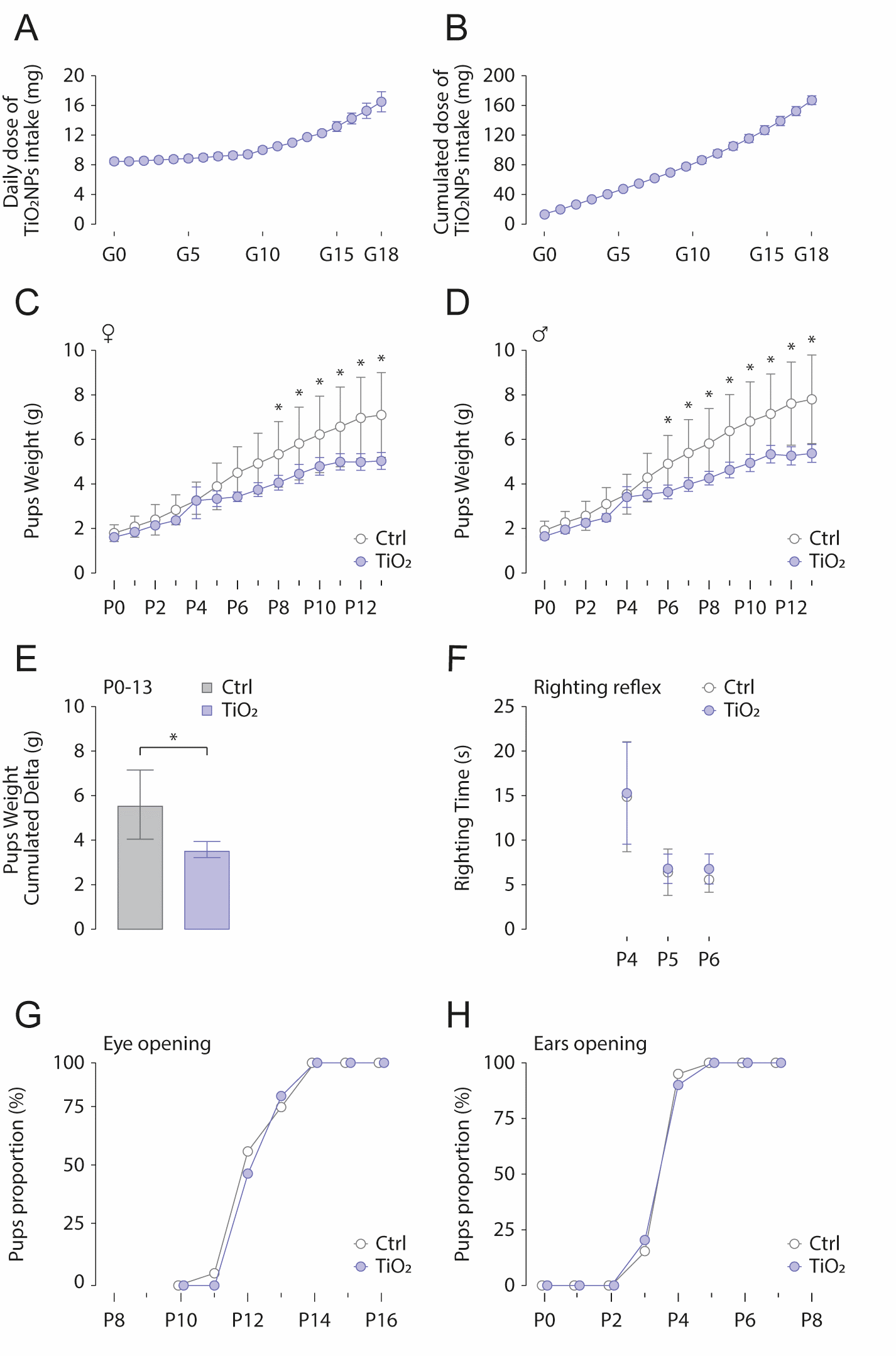


**Figure S1: Developmental milestones in TiO2NPs-exposed pups.** (**A**) Scatter plot showing the daily dose of TiO2NPs consumed by females during gestation. (**B**) Scatter plot showing the cumulative amount of TiO2NPs consumed by females during gestation. (**C, D**) Scatter plots showing body weight evolution from P0 to P13 in non-exposed and TiO2NPs-exposed female (**C**) and male (**D**) pups (non-exposed females, n = 14; non-exposed males, n = 16; TiO2NPs-exposed females, n = 12; TiO2NPs-exposed males, n = 12). (**E**) Bar chart illustrating cumulative weight gain over the P0-P13 period in non-exposed (n = 30) and TiO2NPs-exposed (n = 24) pups. (**F**) Scatter plot showing the time required to perform the righting test in non-exposed (n = 15) and TiO2NPs-exposed (n = 15) pups. (**G, H**) Scatter plots illustrating the progression of eye (**G**) and ear (**H**) opening from P0 to P16 in non-exposed (n = 15) and TiO2NPs-exposed (n = 15) pups.


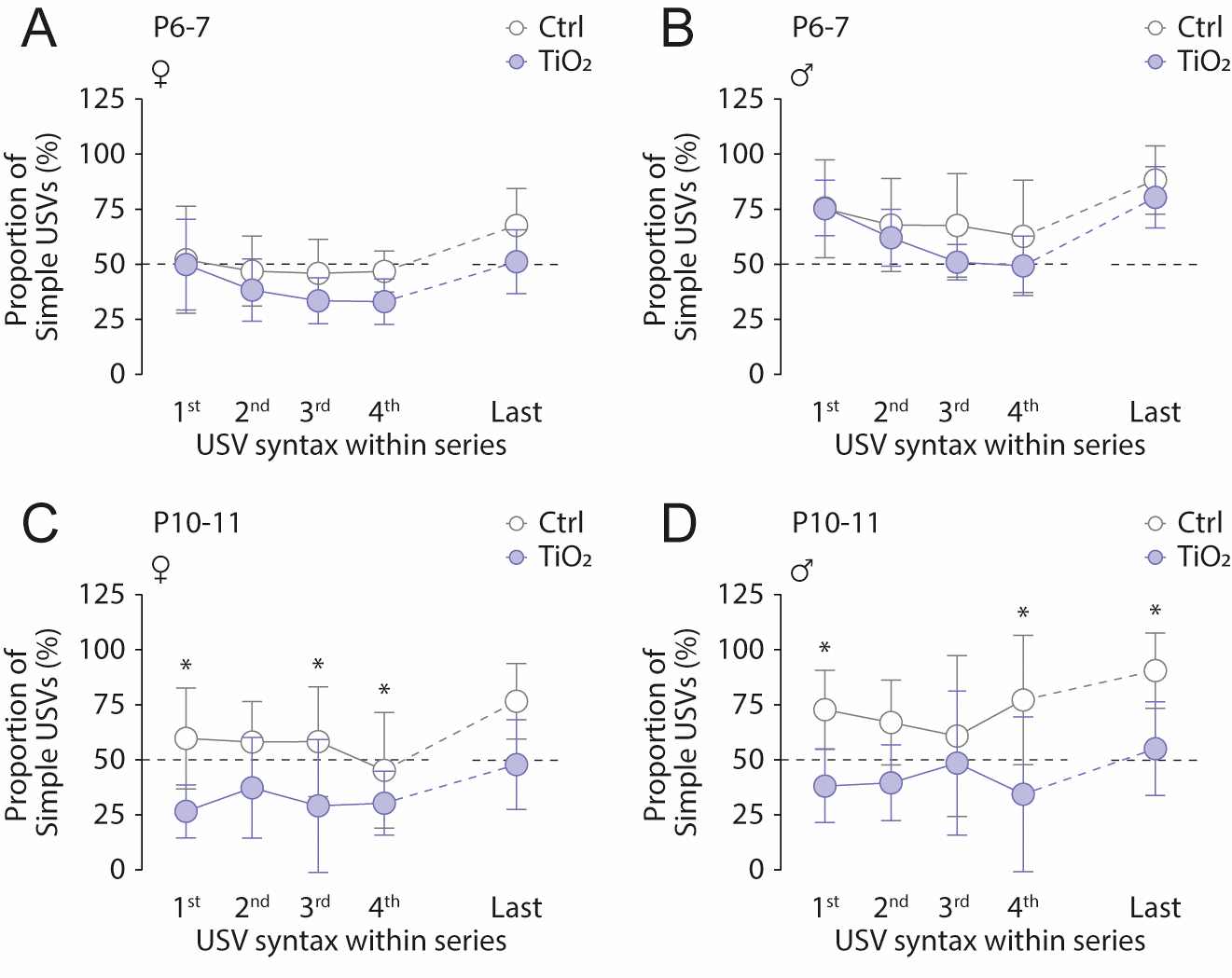


**Figure S2: TiO2NP exposure affects USV series syntax development over the P2-P13 period** (related to Fig. 6)**.** (**A, B**) Scatter plots of mean syntax use in P4-5 female (**A**) and male (**B**) pups. (**C, D**) Scatter plots of mean syntax use in P10-11 female (**C**) and male (**D**) pups. Number of female Control pups: 12; Number of male Control pups: 10; Number of female TiO2NPs pups: 12; Number of male TiO2NPs pups: 12.


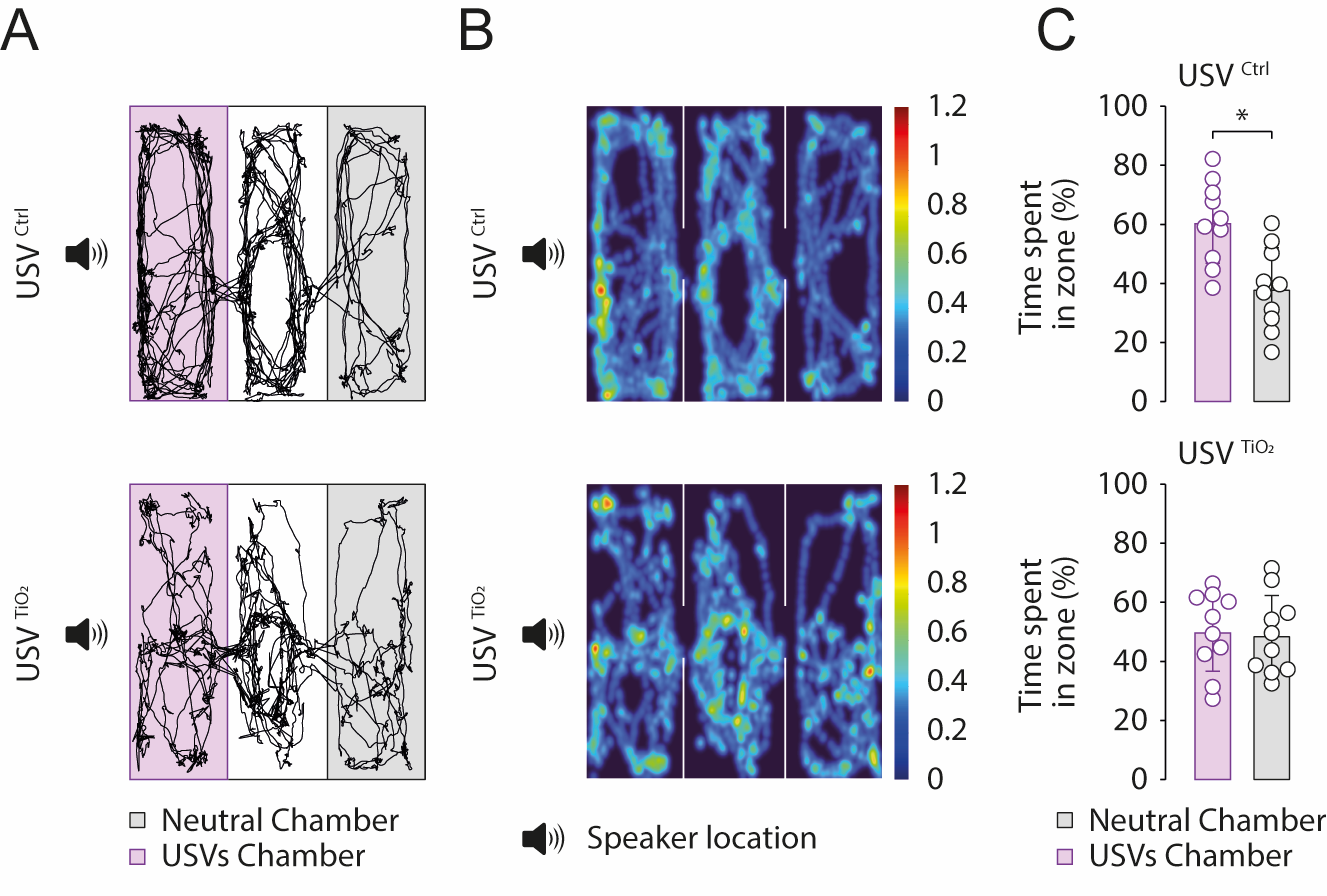


**Figure S3: USVs from TiO2NPs-exposed pups fail to elicit maternal guidance in exposed dams.** (**A**) Schematic representation of the playback experiment protocol. The USV chamber (speaker location) is shaded in light pink, and the neutral chamber is in light gray. Black traces indicate the movement of a TiO2NPs-exposed adult female when USVs from control (upper) or TiO2NPS-exposed pups (middle) were played. (**B**) Heat map highlighting preferred stopping zones (in red) of adult females during the test. (**C**) Bar charts showing the time spent by adult females in the USV chamber (light pink bars) vs. the neutral zone (light gray bars) when presented with USVs from control (USV^CTRL^) or TiO2NPs-exposed (USV^TiO2^) pups. Number of exposed females: USV^CTRL^: 10; USV^TiO2^: 10.
